# Self‐Regulative Nanogelator Solid Electrolyte: A New Option to Improve the Safety of Lithium Battery

**DOI:** 10.1002/advs.201500306

**Published:** 2015-11-18

**Authors:** Feng Wu, Nan Chen, Renjie Chen, Qizhen Zhu, Guoqiang Tan, Li Li

**Affiliations:** ^1^ Beijing Key Laboratory of Environmental Science and EngineeringSchool of Materials Science and EngineeringBeijing Institute of TechnologyBeijing100081P.R. China; ^2^ Collaborative Innovation Center of Electric Vehicles in BeijingBeijing100081P. R. China

**Keywords:** ionic liquid, lithium battery technology, nanogelators, solid electrolyte

## Abstract

The lack of suitable nonflammable electrolytes has delayed battery application in electric vehicles. A new approach to improve the safety performance for lithium battery is proposed here. This technology is based on a nanogelator‐based solid electrolyte made of porous oxides and an ionic liquid. The electrolyte is fabricated using an in situ method and the porous oxides serve as a nonflammable “nanogelator” that spontaneously immobilizes the ionic liquid. The electrolyte exhibits a high liquid‐like apparent ionic conductivity of 2.93 × 10^−3^ S cm^−1^ at room temperature. The results show that the nanogelator, which possess self‐regulating ability, is able to immobilize imidazolium‐, pyrrolidinium‐, or piperidinium‐based ionic liquids, simply by adjusting the ion transport channels. Our prototype batteries made of Ti‐nanogeltor solid electrolyte outperform conventional lithium batteries made using ionic liquid and commercial organic liquid electrolytes.

## 1 Introduction

Electrochemical storage has attracted a great deal of attention as power storage system for contemporary and emerging technologies,^1^, ^2^, ^3^, ^4^ such as smart phone, intelligent robot, unmanned aerial vehicle, and electric vehicles. Usually these electronic devices need to be used in extreme environment (low or high temperature). As everyone knows, batteries may trigger fires or explosions when exposed to certain undesirable conditions.^5^, ^6^ This issue can be circumvented through the use of solid—that is, glass, ceramic or polymer‐based—electrolytes. However, the applications of such electrolytes are limited due to moderate ionic conductivity. Ionic liquid electrolytes (ILEs) are considered a potential means of improving the safety of lithium‐ion batteries.^7^, ^8^ Liquid state of ILEs require separators for mechanical stability, thus introduce several constraints on cell safety and design. Most research has focused on immobilizing ILEs by impregnating them with polymeric materials such as poly(ethylene oxide) and poly(acrylonitrile).^9^, ^10^, ^11^ Actually, safety issues are not completely eliminated owing to the intrinsic flammability of polymer‐based materials.

**Figure 1:**
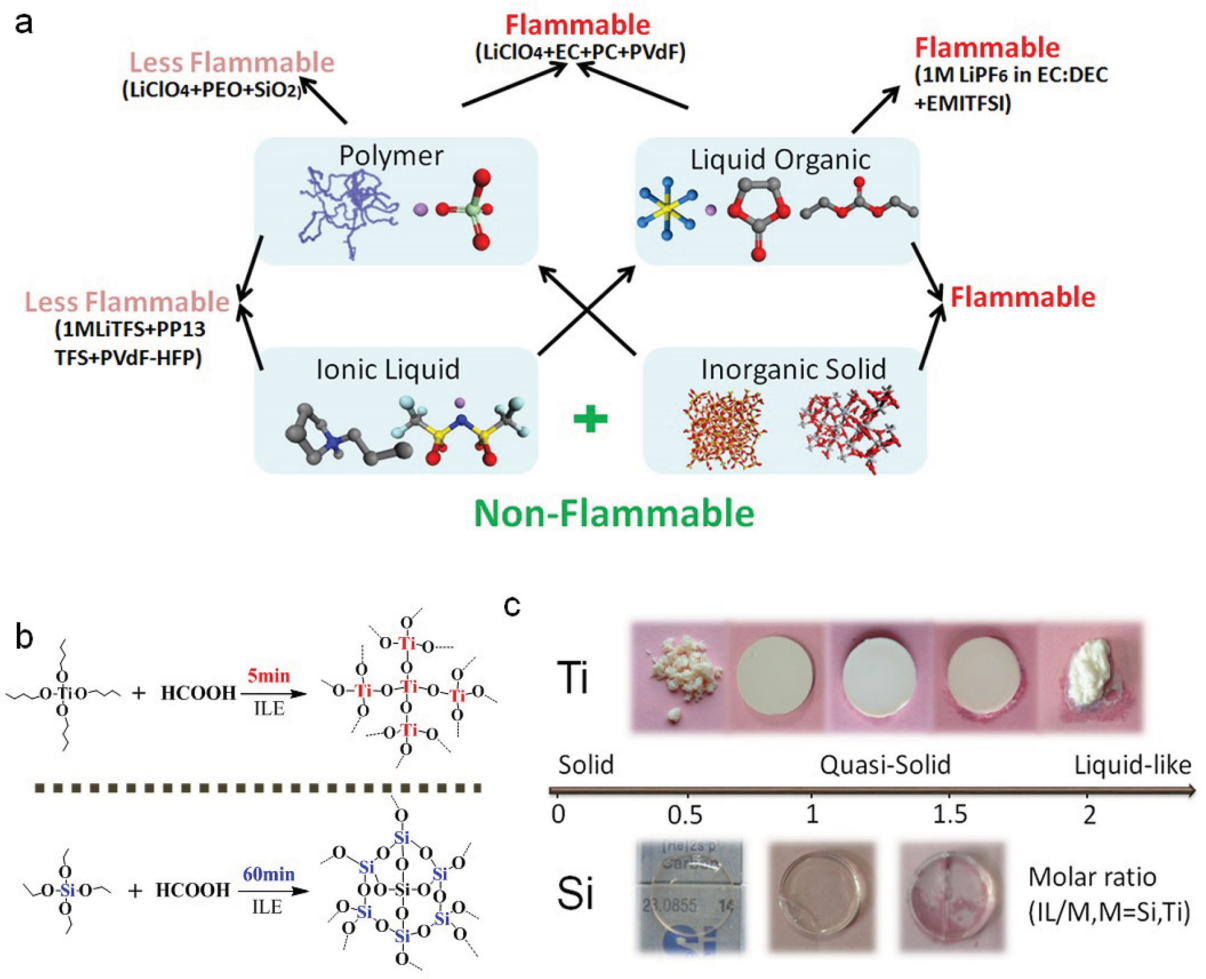
a) Existing electrolytes are made from four basic electrolyte materials: polymers, organic liquids, ionic liquids, and inorganic solids. Of these, only ionic liquids and inorganic solids are nonflammable. b) Ti‐nanogelator and Si‐nanogelator monolith synthesis. c) Photographs of the solid, quasi‐solid, and liquid‐like phase electrolytes with various molar compositions of IL and M (M = Si, Ti).

An alternative strategy that overcomes the shortcomings is to adopt inorganic oxide particles as host to hold the ILE in position.^12^, ^13^, ^14^, ^15^, ^16^, ^17^, ^18^, ^19^, ^20^, ^21^, ^22^ We introduce a unique class of solid‐like electrolytes, nanogelator–based electrolytes, consisting of ionic liquid salt solution and inorganic insulated matrix. The concept of “nanogelator” was originally reported by Matsusaki. He proposed the multiarmed poly (ethylene glycol) terminally modified with collagen mimetic peptide nanoparticles, ca. 25 nm in diameter, acting as nanogelators for collagen gels.^23^ A nanogelator is defined as an inorganic curing agent, which possesses solid interconnected network structure. It is used to immobilize ionic liquids. In this work, inorganic insulated matrix with porous structure is described as nanogelator. In situ hydrolysis and condensation situation, the nanogelator is able to spontaneously immobilize the ionic liquid. The risk of fire or explosion is eliminated because the inorganic host is nonflammable (**Figure** **1**a) and can hold the liquid electrolyte stably in its pores. The nanogelator with self‐regulating ability can adapt to different kinds of ionic liquids by adjusting the ion transport channel structure. The resulting electrolyte combines the advantages of both the inorganic oxide matrix (with mechanical stability, low flammability, and optical transparency) and the ILE (with high ionic conductivity, as well as thermal and electrochemical stability). Titanium dioxide (TiO_2_) has attracted great attention due to its low toxicity, good thermal stability, high temperature decomposition, and nonvolatile. Hence, TiO_2_ is believed to be a potential candidate as nanogelator to confine ILE. The TiO_2_‐based nanogelator electrolyte for lithium batteries presented here is first reported, which shows high discharge capacity and stable cycle performance.

## 2 Results and Discussion

### 2.1 Electrolyte Preparation

Four ionic liquids were investigated and tested for preparation of TiO_2_‐based nanogelator electrolytes (referred to as TiSEs, from here on (Table S1, Supporting Information)). TiSEs were prepared using a straightforward nonhydrolytic sol–gel method.^24^ In brief, the titanium alkoxide precursor was added to a solution of LiTFSI in ionic liquid. When the solution was mixed with formic acid, gel networks were immediately obtained within 5 min (Figure 1b). For the sake of comparison, the gel time of the SiO_2_‐based electrolyte, namely SiO_2_‐based nanogelator solid electrolytes (referred to as SiSEs, from here on), was found to be required in excess of 1 h,^25^ suggesting that the formation rate of the titanium–oxygen linkages is much faster than that of the silicon–oxygen linkages in a formic acid environment. The TiSEs could be used as “paint” in the future, once two liquids mixed, it can fast spray or ink jet printings onto the electrodes of any size and shape. Therefore, the electrolyte can easily be in situ applied in lithium battery fabrications.

### 2.2 Nanogelator/Electrolyte Morphology

In the IL:nanogelator molar ratios range of 0–2, the TiSEs can be categorized into three phases: solid, quasi‐solid, and liquid‐like (Figure 1c). The samples with low IL content (i.e., IL:Ti‐nanogelator = 0–0.5) are in the solid phase and are rigid and fragile. The quasi‐solid phase samples, synthesized with a molar composition ratio of IL:Ti‐nanogelator = 0.5–1.5, are smooth, nonexuding, dimensionally stable, homogeneous, crack‐free, and slightly flexible. They produced approximately 82–88 wt% of ionic liquid electrolyte. The samples with high IL content (IL:Ti‐nanogelator = 1.5–2) represent a liquid‐like phase and exhibited liquid overflow after 6 months of storage.

**Figure 2:**
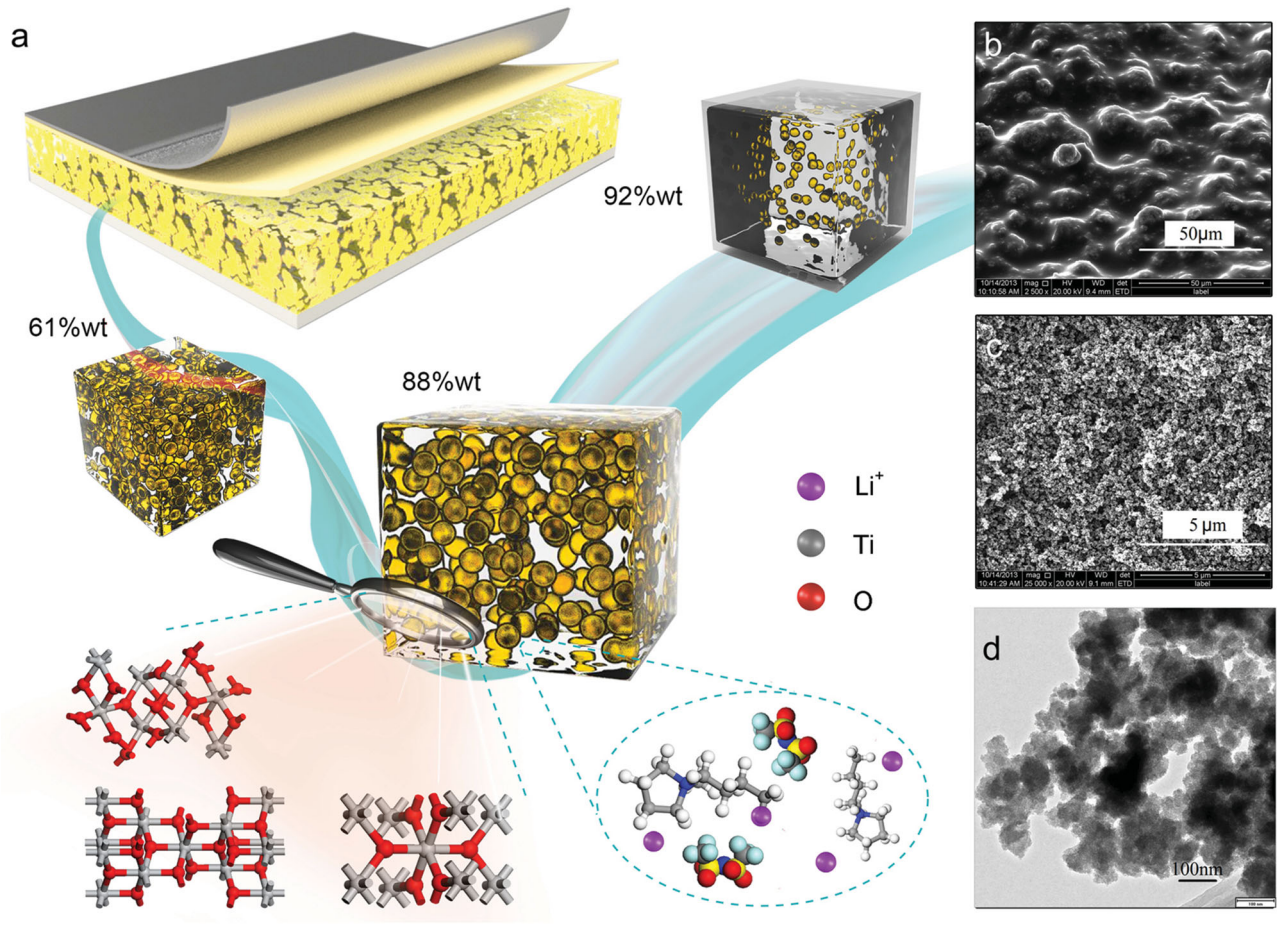
a) Proposed schematic structure of nanogelator‐based solid electrolyte with various ionic liquid electrolyte mass fractions. The inorganic oxide is shown as yellow balls and the ionic liquid electrolyte penetrating the inorganic oxide matrix is represented in white. b) SEM images of the TiSE‐2 with 88 wt% ionic liquid electrolyte content. c) SEM images of the cross‐section of the TiSE‐2 after removal of the ionic liquid. d) TEM image of mesoporous Ti‐nanogelator matrix after removal of the ionic liquid.

In the quasi‐solid phase, Ionic liquid was trapped as an interconnected liquid film coated on the surface of the nanoparticles (see **Figure** **2**a,b). Each ionic liquid electrolyte was extracted from the TiSEs using an acetonitrile solvent, leaving behind the Ti‐nanogelator matrices. The Ti‐nanogelator matrix had a porous structure, with particles ranging from 5 to 60 nm (Figure 2c). The TEM image (Figure 2d) shows aggregates of Ti‐nanogelator particles that formed 3D, continuous, and nanostructured ion transport channels. The cations of ILs were less than 1 nm in diameter and the TFSI^−^ ion was 0.91 nm in the longest dimension.^26^ Given that these ions were smaller than the pore diameter of the network, we propose that the ionic liquid acts as a template solvent and is incorporated into the matrix of the Ti‐nanogelator on the molecular scale through a self‐assembly process. Energy dispersive spectroscopy (Figure S1, Supporting Information) showed that the chemical formula that best characterizes the Ti‐nanogelator is one titanium atom with two oxygen atoms.

**Figure 3:**
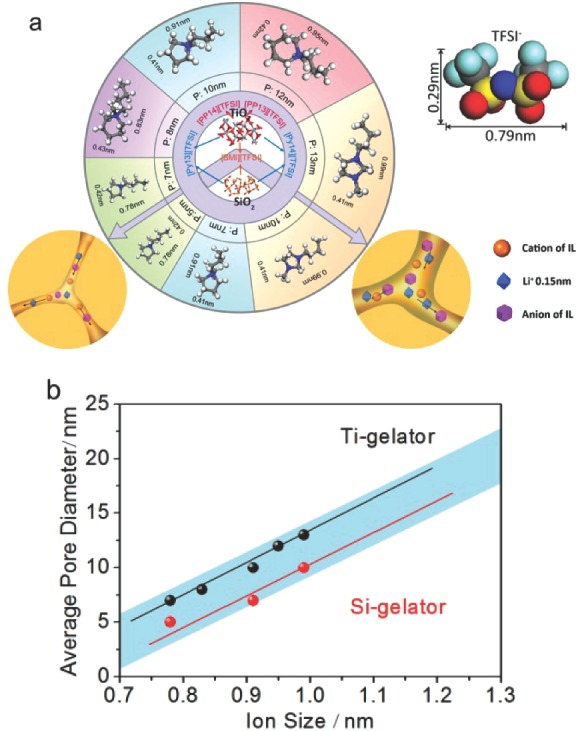
a) The relationship between the size of the ions in the ionic liquid and the pore diameter of the ion transport channels. b) Plot of the ion size versus the average pore diameter for both nanogelator systems studied.

**Figure** **3**a shows the relationship between the size of the ions in the ionic liquid and the pore diameter of the network. We next optimized and calculated the ion sizes of ionic liquid using Materials Studio 5.5. For the Ti‐nanogelator, the Barrett–Joyner–Halenda pore diameter increases on average from 7 to 13 nm when the cationic size of the ionic liquid (calculated along the longest expansion) increases from 0.78 to 0.99 nm. The Si‐nanogelator follows the same trend: samples prepared from an ionic liquid with small ions have a small pore diameter, while samples prepared from an ionic liquid with large ions have a large pore diameter. For each nanogelator electrolyte, the average pore diameter increases nearly linearly with increasing ionic liquid ion size (Figure 3b). These results suggest that the average pore diameter of the ion transport channels can be tuned by varying the sizes of the ions within a given IL.

### 2.3 Structural Properties

To determine whether confinement within a porous matrix has any effect on the structure of an ionic liquid, we performed X‐ray diffraction (XRD), Fourier transform infrared (FTIR) spectroscopy, and nuclear magnetic resonance (NMR) on both confined and unconfined samples. The XRD spectra (Figure S2, Supporting Information) of the Ti‐nanogelator matrix were found to be typical of amorphous solids. The FTIR spectra of unconfined and confined ILEs (Figure S3, Supporting Information) were virtually identical, suggesting that no direct chemical bonding occurred between the nanogelator and the ionic liquid. Thus we can deduce that ILEs were not affected by confinement within the nanogelator.

**Figure 4:**
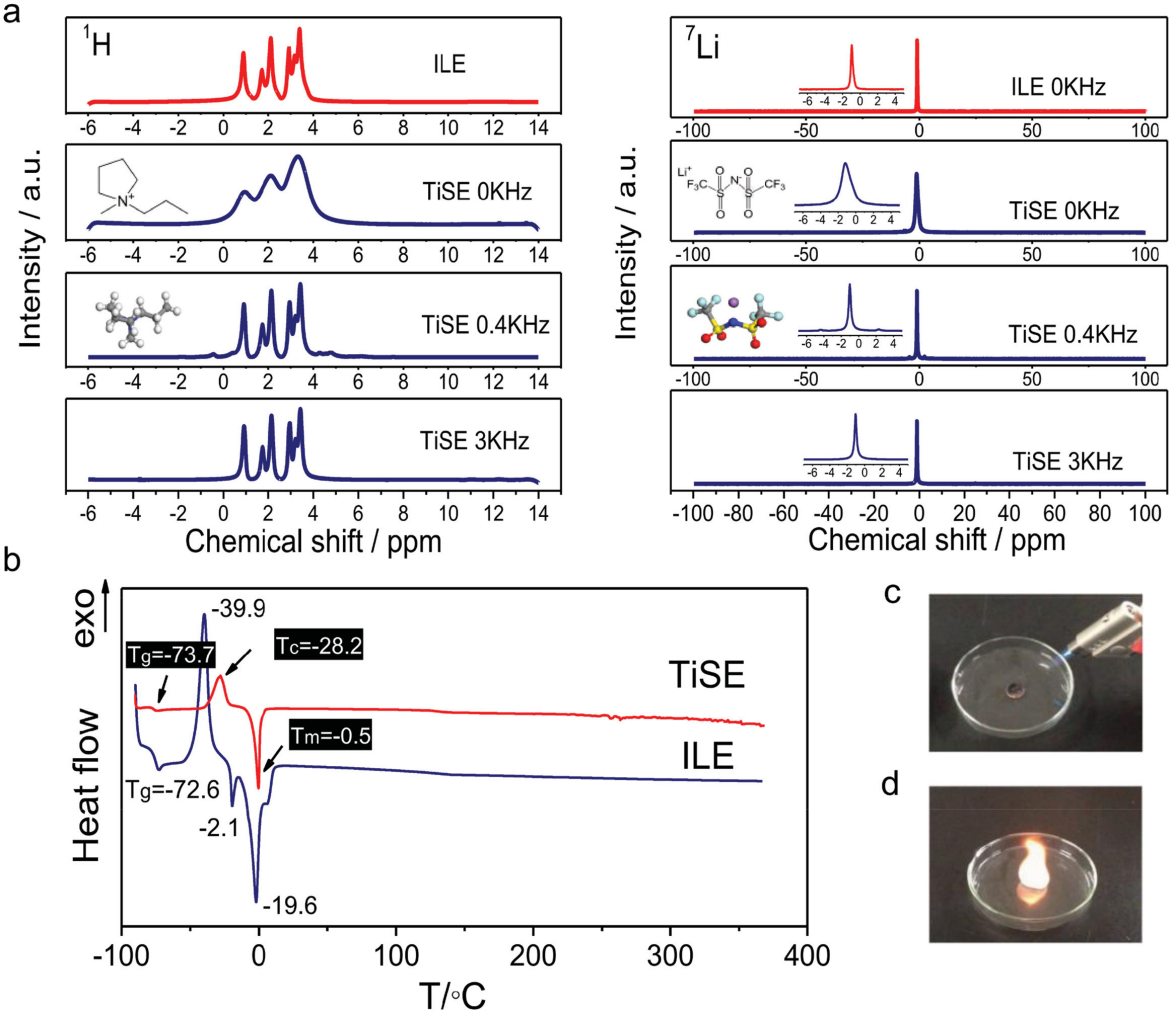
a) Room‐temperature liquid‐state ^1^H NMR and ^7^Li NMR spectra of the ILE 1 m LiTFSI‐Py13TFSI and solid‐state ^1^H MAS NMR and ^7^Li NMR of TiSE‐1 at different spinning rates (0, 0.4, and 3 KHz). b) Differential scanning calorimetry thermograms of ILE and TiSE‐1. Flammability tests of c) TiSE‐1 and d) the 1.0 m LiPF_6_‐EC/DEC (1:1 by volume) commercial electrolyte using a Bunsen burner.

We next examined the flow dynamics of pristine ILE and of TiSE with an IL:Ti ratio of 1.25 using solid‐state ^1^H and ^7^Li MAS NMR (**Figure** **4**a). We detected both ^1^H and ^7^Li, corresponding to the Py13^+^ and Li^+^ components of the Py13TFSI and LiTFSI. For TiSE, some resolution in the ^1^H and ^7^Li spectra is visible as a broad peak without rotation (0 kHz), and so the resolution could be recovered as the spinning rate is raised to 0.4 kHz. These results show that the dynamics of the confined ionic liquid experienced only a slight slowing down. The ^1^H NMR spectra for TiSEs are similar to the spectra for ILEs, showing no signals related to formic acid or to Ti‐OC_4_H_9_ and Ti‐OH groups; this indicates that titanium alkoxide had completely transformed into TiO_2_. The signals become significantly narrower, and no narrowing of the line occurs as the spinning rate increases, indicating that chemical shift anisotropy and dipolar interactions are averaged in confined ionic liquids.^27^ Thus we can conclude that in our samples, the mobility of confined ILEs was close to the mobility of liquid‐state ILEs.^28^ This finding could be interesting for various applications such as electrolytes for lithium‐ion batteries, lithium–sulfur batteries and lithium–air batteries.

Differential scanning calorimetry (DSC) traces for ILE and TiSE are depicted in Figure 4b. The glass transition temperature (*T*_g_), the exothermic cold crystallization (*T*_c_) and the endothermic melting temperature (*T*_m_) of the ILE change upon confinement. This could be due to strong interactions between the Ti‐nanogelator wall and the confined ionic liquid.^29^, ^30^ The confinement of ILE in TiSE exhibits the same stability at high temperatures as the pristine ILE; in both cases, no endothermic or exothermic reactions were observed over the temperature range from room temperature to 375 °C, indicating that the TiSE electrolyte had good thermal stability and may be suitable for use in a high‐temperature environment. Flammability tests, as expected, the TiSE did not exhibit any combustion, even after 60 s of ignition with a flame source where the outer flame temperature is up to 1300 °C, indicating that this type of solid electrolyte is nonflammable. However, the commercial electrolyte (1.0 m LiPF_6_‐EC/DEC (1:1 in volume)) was highly flammable and easily ignited once in contact with the flame.

### 2.4 Electrochemical Tests

**Figure 5:**
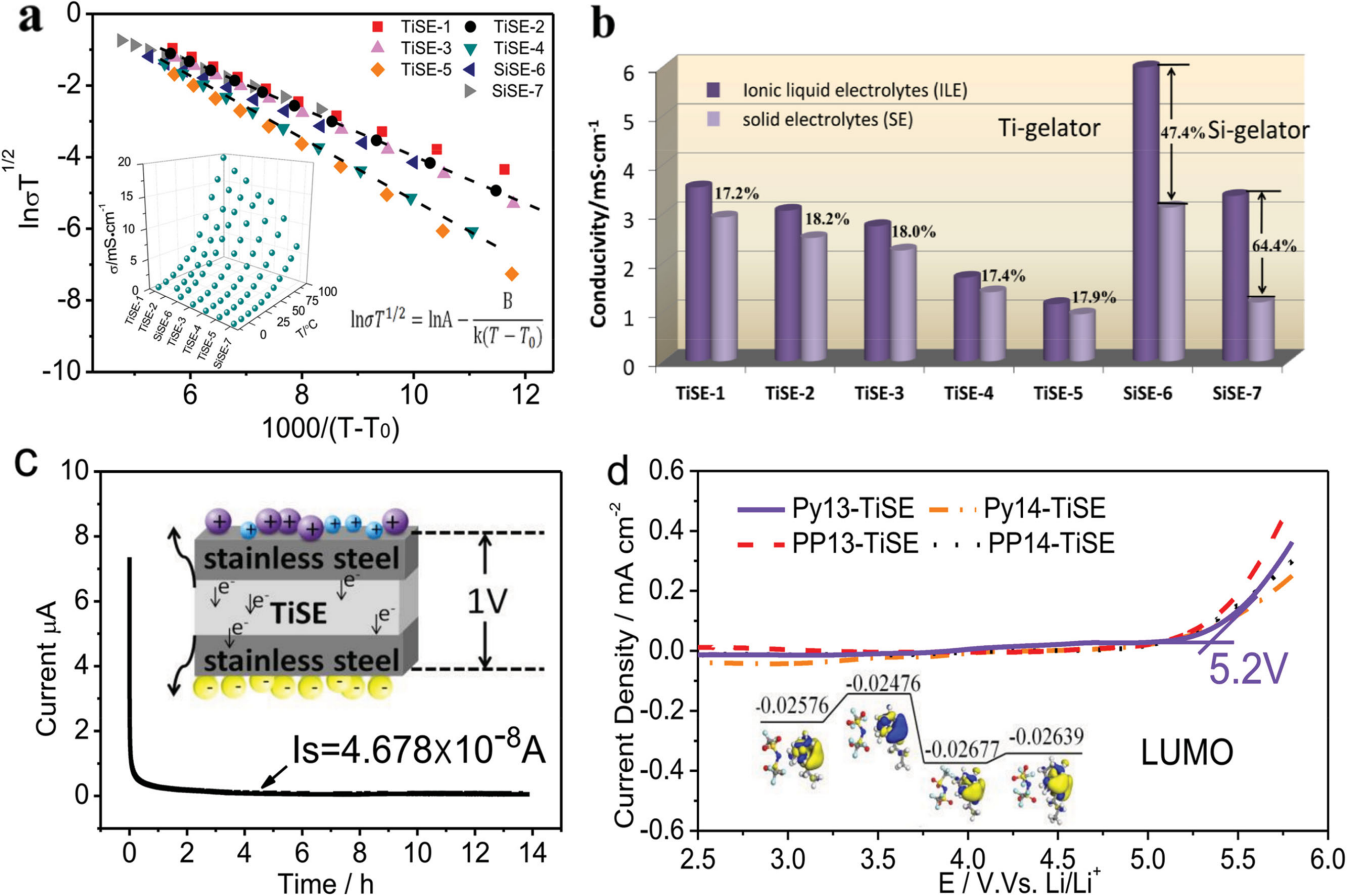
a) Conductivity VTF plots of the TiSE and SiSE samples. Parameters used in the VFT fits are provided in Table S4 (Supporting Information). Inset: conductivity as a function of temperature. The working temperature was raised from ambient temperature to 100 °C and all cells were cooled down to ambient temperature followed by cooling down to −10 °C. b) Ionic conductivity of the ILE before and after confinement in a Ti‐nanogelator and Si‐nanogelator. c) Electronic conductivity of the TiSE‐1 sample. d) Linear voltammetry plots of the Py13‐TiSE, Py14‐TiSE, PP13‐TiSE, and PP14‐TiSE at room temperature.

Low electronic conductivity and high ionic conductivity are fundamental requirements for electrolytes intended for use in electrochemical devices. The calculated electronic conductivity of TiSE is 2.1 × 10^−9^ S cm^−1^ (**Figure** **5**c). High ionic conductivity can be obtained by introducing more IL into the nanogelator‐based electrolytes. This is usually accompanied by reduced dimensional stability and mechanical strength. A weak mechanical strength nanogelator‐based electrolyte can easily cause a short circuit between a cathode and an anode when it is applied in practical applications. Thus, a quasi‐state phase with a molar ratio of IL/Ti = 1.25 was selected in this study, which has high IL content and acceptable mechanical strength.

Over temperatures ranging from −10 to 100 °C, the TiSEs and SiSEs followed a well‐behaved VFT relationship (*σ* = *AT* ^−0.5^ exp(−*B*/(*T*−*T*_0_)); the parameters *A*, *B,* and *T*_0_ are provided in Table S4 in the Supporting Information). Ion transport in the solid electrolyte was reversible; there was no change in conductivity after reducing the temperature from 100 °C, indicating that solid electrolytes are likely to withstand a temperature of 100 °C without affecting their transport properties. The highest conductivity of 7.7 × 10^−4^ S cm^−1^ at 0 °C, 2.74 × 10^−3^ S cm^−1^ at 25 °C and 2.3 × 10^−2^ S cm^−1^ at 100°C was obtained for the sample TiSE‐1 (Figure S4 and Table S3, Supporting Information).

Ti‐nanogelator solid electrolyte demonstrates significantly improved ionic conductivity compared to Si‐nanogelator solid electrolytes (Figure 5b). The conductivity of the SiSE system decreased dramatically (by 40%–70%) after the ILE was confined; the conductivity values of the TiSE, in contrast, decreased by only 20%. Overall, an optimized low Ea of 0.12 eV was obtained in the TiSE‐1 sample, which is much lower than the Ea of the SiSEs (Table S1, Supporting Information), suggesting that the Ti‐nanogelator‐based solid electrolyte underwent faster ion transport than the Si‐nanogelator‐based solid electrolytes. We conclude that an appropriate environment for Li^+^ migration exists in TiSEs, which promotes the dissociation of LiTFSI salts and ionic liquids where the Ti‐nanogelator acts as the additional dielectric substance.^31^

Electronic conductivity was determined at room temperature using potentiostatic coulometry measurements (Figure 5c), run on a symmetric stainless steel (SS) ion‐blocking electrode SS/TiSE pellet sample/SS cell and applying a 1 V polarization (*U*). Under given cell conditions, the ion flow decreased with time due to the pile‐up of ions at the SS electrode. The electronic flow remained constant during the experiment as a result of the blocking effect of the electrodes.^32^ The current response of the system achieved a steady state after 6 h, with a value of 4.68 × 10^−8^ A, in which the conductivity is considered to be mostly the result of electron flow. The electrical conductivity of the solid electrolyte is calculated using *ϒ* = Is*L*/*US*, where the Is is standing current, *S* = 2.1124 cm^2^ and *L* = 0.0944 cm are the surface area and the thickness of the TiSE monolith, respectively. The calculated electronic conductivity is 2.1 × 10^−9^ S cm^−1^ (i.e., approximately six orders of magnitude lower than the ionic conductivity).

Figure 5d presents linear sweep voltammograms of a platinum electrode with all electrolytes in different ionic liquids, extended to the anodic side. It shows that all the samples had great anodic stability. To our surprise, the presence of a Ti‐nanogelator in one of the ILEs did not significantly affect the oxidation potential; for all cases the potential exceeded 5 V (vs Li/Li^+^). Comparing the four plots shows that the Py13‐TiSE is electrochemically more stable than the other TiSEs tested, and its oxidation potential is found to be 5.2 V (vs Li/Li^+^). This value is acceptable for use in practical high‐voltage lithium batteries.

### 2.5 Cells Performance

**Figure 6:**
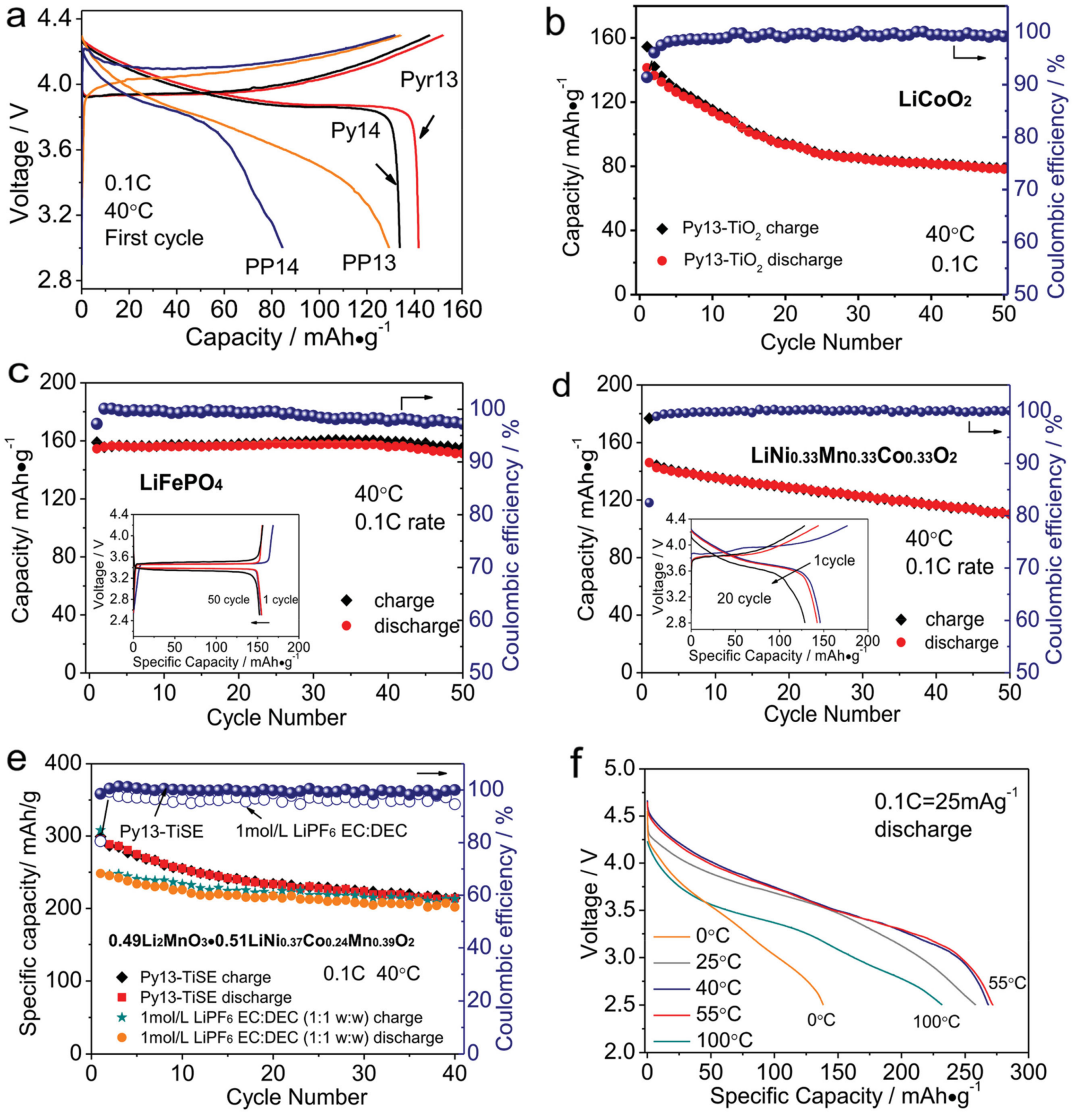
a) First charge–discharge curves of Li/TiSE/LiCoO_2_ cells. b) Cycling performance of the Li/Py13–TiSE/LiCoO_2_ cell. Cycling performance of c) Li/Py13‐TiSE/LiFePO_4_ and d) Li/Py13‐TiSE/LiNi_0.33_Mn_0.33_Co_0.33_O_2_, insets indicate charge and discharge profiles. e) Charge–discharge cycle performance curves of Li/Py13‐TiSE/lithium‐rich and Li/LiPF_6_‐EC‐DEC/lithium‐rich cells. f) Voltage–capacity plots of Li/Py13‐TiSE/lithium‐rich cell at 0, 10, 25, 40, 55, and 100 °C.

We assembled solid‐state Li/TiSE/LiCoO_2_ foil half‐cells with no extra separator to evaluate the suitability of those electrolytes for use in lithium‐ion batteries. The initial voltage–capacity profiles of the devices are displayed in **Figure** **6**a. Remarkably, the best performance was shown by the Py13‐TiSE. The discharge capacity of Py13‐TiSE was found to be 141.7 mAhg^−1^ at 0.1 C. These results suggest that Py13‐TiSE is indeed battery‐active and cathode‐compatible. We selected Py13‐TiSE for further study. The CV curve of Li/Py13‐TiSE/LiCoO_2_ shows two pairs of oxidation and reduction peaks (Figure S5a, Supporting Information). The discharge capacity and Coulombic efficiency of the cell are 115.5 mAhg^−1^ and 98.7% after 10 cycles (Figure 6b).

Coin cells prepared with LiFePO_4_ cathode (Figure 6c) were galvanostatically charged and discharged at 40 °C. The initial discharge capacity of the Li/Py13‐TiSE/LiFePO_4_ cell was 154.5 mAhg^−1^ at 1/10 C; this then increased with cycle number and stabilized at 156 mAhg^−1^, which is higher than the discharge capacity observed in LiTFSI‐Py13TFSI ionic liquid electrolyte (132 mAhg^−1^ at 1/15 C).^33^ The Coulombic efficiency of the electrolyte was over 98%. The CV curve (Figure S5b, Supporting Information) shows a pair of oxidation and reduction peaks corresponding to lithium extraction/insertion in the LiFePO_4_/FePO_4_ structure. At 40 °C, the polarization potential was 0.08 V, which explains why the cell operates well.

Li/Py13‐TiSE/LiNi_0.33_Mn_0.33_Co_0.33_O_2_ half‐cells deliver specific capacities as high as 146 mAhg^−1^ in the beginning of the first cycle and 110.2 mAhg^−1^ after the 50th cycle between 2.8 and 4.3 V (Figure 6d). Cycling between 2.8 and 4.2 V resulted in excellent capacity retention and good charge–discharge capacities of greater than 120 mAhg^−1^, which is much higher than cells that use the SiSEs (105 mAhg^−1^ after the tenth cycle).^34^ We attribute this improvement to the better compatibility between LiNi_0.33_Mn_0.33_Co_0.33_O_2_ and the Ti‐nanogelator interface, which makes Li^+^ intercalation and de‐intercalation easier. The Coulombic efficiency, apart from the initial irreversible capacity, levels off at 100% at the start of the second cycle. CV measurements showed an oxidation peak at 3.81 V and reduction peak at 3.50 V (Figure S5c, Supporting Information). Cycling is possible up to 4.5 V versus Li/Li^+^ (Figure S6, Supporting Information), which delivers an enhanced capacity of approximately 150 mAhg^−1^.

The nanogelator electrolyte performing well with decomposition up to voltage of about 5.2 V versus Li/Li^+^, a half cell with a 0.49Li_2_MnO_3_·0.51LiNi_0.37_Co_0.24_Mn_0.39_O_2_ (lithium‐rich) high voltage spinel has been assembled. The discharge capacity obtained with the solid Py13‐TiSE (295 mAhg^−1^) is higher than obtained with the liquid 1.0 m LiPF_6_ ‐EC/DEC electrolyte (248.2 mAhg^−1^); the discharge capacity of the Py13‐TiSE returns to 223.5 mAhg^−1^ after 30 cycles (Figure 6e). This demonstrates that the structure of the composite solid electrolyte was stable at high voltage. The CV curve (Figure S5d, Supporting Information) shows a pair of well‐defined oxidation–reduction peaks and suggests that lithium‐rich can be very well charged and discharged in the Py13‐TiSE. We studied impedance properties to gain a better understanding of the effect of electrolyte composition on electrochemical performance. Figure S7 (Supporting Information) shows the impedance plots of Li/TiSE/lithium‐rich cells before cycling and after 30 cycles at 0.1 C. One semicircle in the high‐frequency region and Warburg‐type impedance in the low‐frequency region can be observed in both impedance plots. The interface impedance increased after 30 cycles and the interface state changed continually, corresponding to the constant chemical reaction between the solid electrolyte and the electrode, which forms the passivating film with resistance *R*_film_.

An important property of a battery is its ability to work well over a wide temperature range. To this end, we tested the performance of the Li/Py13‐TiSE/lithium‐rich cell over the range 0–100 °C. We found that the capacities measured at 55 °C were higher than the corresponding values obtained at other temperatures (Figure 6f). The cell performed poorly at 0 °C, with the voltage platform declined rapidly. This poor performance can be attributed to the thermal dependence of the lithium ion diffusion in both electrolyte and lithium‐rich cathode. However, the discharge capacity of the cell is still 130 mAhg^−1^, at a value which is considerably higher than that offered by any solid lithium ion battery.^35^ On the other end of the temperature range at 100 °C, we observed that the cells operated with a discharge capacity of 231 mAhg^−1^. However, the discharge curve is different from the curve obtained at 55 °C. The discharge process at 100 °C consisted of three parts: a steep voltage ramp and two long smooth slopes, which demonstrated that the discharge process essentially changed when the test temperature was increased. To the best of our knowledge, the materials reported here are among the first to operate successfully at such a high temperature. Further studies need to be carried out to clarify the reaction mechanism.

**Figure 7:**
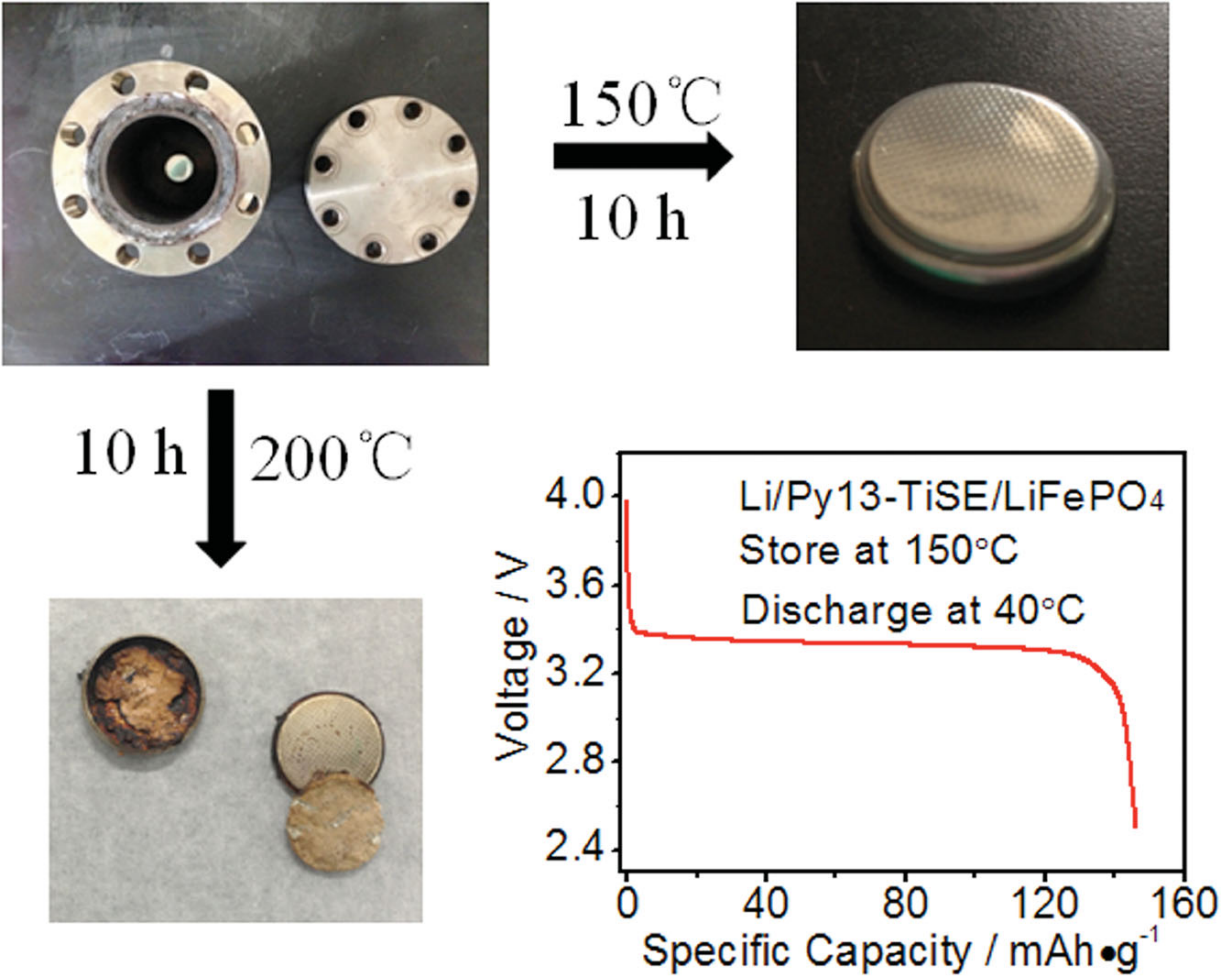
High‐temperature storage test of TiSE‐based cell.

In addition, the high temperature storage test was carried out for Li/Py13‐TiSE/LiFePO_4_ cell (**Figure** **7**). The assembled cells were charged for 100% SOC and then stored in an oven for 10 h at 150 and 200 °C, respectively. After that, the full charged cells were discharged at 40°C. The cell stored at 150°C shows damage free, retaining more than 92% of initial capacity. While the cell store at 200°C explode, the positive and negative shells are totally separate. The charged LiFePO_4_ has a high onset temperature of 250 °C and exotherm peaks at 280 and 315 °C on the DSC profile.^36^ The TiSE shows good thermal stability, no endothermic and exothermic reaction between 25 and 350°C. Thus, LiFePO_4_ and TiSE are not the main factor for the explosion of the cell at elevated temperature. Meanwhile, the melting temperature of lithium metal is 180°C, thus the lithium anode is the key factor to the thermal runaway.

## 3 Conclusions

In summary, immobilizing ionic liquids within an inorganic solid matrix hold substantial promise for large‐scale lithium batteries applications by virtue of nonflammability. The idea also provides direction for the future study of safety electrolytes. The self‐regulating characteristics of nanogelator should also be used to prepare tunable pore diameter of catalyst, ceramic materials and drug‐delivery carriers. The sol–gel approach is an efficient and low cost approach to synthesis Ti‐nanogelator. Thanks to the short gel time, the ink‐jet printing technology can be used to mass production in the future. Those features will make nanogelator‐based solid electrolytes strong forerunners in the race to replace organic solvents in lithium‐ion batteries, Na^+^ ion batteries, supercapacitors, solar cells and fuel cells. In this race however there are still numerous hurdles to be overcome, additional work is certainly needed to further improve the properties of these batteries, especially in terms of cycle life, interfacial compatibility with electrodes, and reaction mechanism between electrolyte and nanogelator‐based electrolyte at high temperature.

## 4 Experimental Section

*Electrolyte Synthesis*: Ti‐based nanogelator solid electrolytes were prepared by nonaqueous self‐assembly sol–gel processing. All of the ionic liquids (purity >99%) were obtained from Shanghai Cheng Jie. A typical example, the LiTFSI (1.37 g, 3 m, 99%), was completely dissolved in Py13TFSI (6.90 g) to form an ionic liquid electrolyte (ILE). Tetrabutyl titanate (4.77 mL, Aldrich, 99%) was added to the ILE and stirred for 20 min. After homogenization, formic acid (4 mL, Aldrich, 98%) was added drop by drop while the mixture was stirred for 1 min. The mixture was then poured into stainless steel molds. Gelation was completed at room temperature over 36 h, after which the molds were dried at 60 °C in a vacuum for 3 d to remove unreacted formic acid and volatile side products. The entire procedure was conducted under an argon atmosphere.

*Material Characterization*: XRD studies were performed using a Rigaku Ultima IVe185 X‐ray diffractometer with a copper Kα radiation source. Morphological studies and element ratio tests were performed on the solid electrolytes using a field emission SEM (Quanata 200f) and TEM (JEM‐2100F) with an accelerating voltage of 200 kV. The pore size distribution for each nanogelator network was evaluated using the Barrett–Joyner–Halenda method^37^ applied to the nitrogen adsorption−desorption isotherm using a Quantachrome NOVA1200e analyzer at 77 K. FTIR spectra were recorded using a Nicolet 6700 FTIR spectrometer. Solid‐state NMR was performed on a Bruker AV 300 spectrometer using tetramethylsilane (SiMe_4_) and lithium carbonate (Li_2_CO_3_) as references. DSC was carried out with a MDC290 analyzer and traces were recorded from −90 to 200 °C at a heating rate of10 K min^−1^.

Flammability tests of both solid and liquid electrolytes (TiSE and commercial electrolyte 1.0 m LiPF_6_‐EC/DEC, respectively) were performed using an electronic Bunsen burner fed with a controlled butane flux that produced an oxidant blue flame. The titanium solid electrolyte was placed in the middle of a Petri dish and heated directly with the Bunsen burner.

*Electrochemical Measurements*: Ionic conductivity measurements were performed by electrochemical impedance spectroscopy using an IM6e electrochemical station (Zahner Elektrik, Germany) over the frequency range between 100 kHz and 10 mHz and with a 5 mV amplitude. Cells were prepared by sandwiching the solid electrolyte samples between two blocking stainless steel electrodes. The working temperature was raised from ambient temperature to 100 °C and all cells were cooled to ambient temperature followed by further cooling to −10 °C. The ionic conductivity was calculated according to *σ* = *L* × *S* ^−1^ × *R* ^−1^, where *σ* is the ionic conductivity, *L* is the distance between the two electrodes, *S* is the geometric area of the electrode/electrolyte interface and *R* is the intercept at the real axis in the impedance Nyquist plot.

LiFePO_4_ (Pulead Technology Industry Company), LiCoO_2_ (Easpring Material), LiNi_0.33_Mn_0.33_Co_0.33_O_2_ (Pulead Technology Industry Company), and lithium‐rich 0.49Li_2_MnO_3_·0.51LiNi_0.37_Co_0.24_Mn_0.39_O_2_ (Argonne National Laboratory, USA) electrode materials were used. The positive electrodes of the lithium half‐cell consisted of 80 wt% cathode material, 10 wt% acetylene black, and 10 wt% polyvinylidene fluoride. The mixture was coated onto aluminum foil and dried at 80 °C in a vacuum overnight. Cycling tests were evaluated in a 2032 coin‐type cell using lithium metal as the anode and the TiSE membrane as a solid electrolytic separator. For the sake of comparison with the liquid electrolyte 1.0 m LiPF_6_‐EC/DEC (1:1 by volume), Celgard 2300 was chosen as a separator. The temperature was controlled using a temperature testing chamber (GDJS‐100, WuxiSuoyate Company).

## Supporting information

As a service to our authors and readers, this journal provides supporting information supplied by the authors. Such materials are peer reviewed and may be re‐organized for online delivery, but are not copy‐edited or typeset. Technical support issues arising from supporting information (other than missing files) should be addressed to the authors.

Supplementary
