# Supplementary material for: Self‐Regulative Nanogelator Solid Electrolyte: A New Option to Improve the Safety of Lithium Battery
Source: Adv Sci (Weinh). 2015 Nov 18;3(1):1500306. doi: 10.1002/advs.201500306 (PMC5063194; doi:10.1002/advs.201500306)
Supplement: Supplementary file 1 — Supplementary [file ADVS-3-0i-s001.pdf]

Copyright WILEY-VCH Verlag GmbH & Co. KGaA, 69469 Weinheim, Germany, 2015.

ADVANCED SCIENCE
Open Access

Supporting Information

for *Adv. Sci.*, DOI: 10.1002/advs. 201500306

Self-Regulative Nanogelator Solid Electrolyte: A New Option to Improve the Safety of Lithium Battery

Feng Wu, Nan Chen, Renjie Chen, Qizhen Zhu, Guoqiang Tan, and Li Li*

Copyright WILEY-VCH Verlag GmbH & Co. KGaA, 69469 Weinheim, Germany, 2013.

Supporting Information

Self-Regulative Nanogelator Solid Electrolyte: A New Option to Improve the Safety of Lithium Battery

Feng Wu, Nan Chen, Renjie Chen, Qizhen Zhu, Guoqiang Tan, Li Li*

Supplementary Table S1 Summary of the nanogelator-based solid electrolytes used in this study.

Sample	Sample compositions (in relative molar ratios)				ILE composition	σ(Sm·cm^{-1})	Ea(eV)	Optical appearance
	TBT	TEOS	HCOOH	ILE		30 °C		
TiSE-1	1	0	7.8	1.25	0.6M LiTFSI/[Py13][TFSI]	2.93	0.12	opaque
TiSE-2	1	0	7.8	1.25	1.0M LiTFSI/[Py13][TFSI]	2.52	0.15	opaque
TiSE-3	1	0	7.8	1.25	1.0M LiTFSI/[Py14][TFSI]	2.26	0.16	opaque
TiSE-4	1	0	7.8	1.25	1.0M LiTFSI/[PP13][TFSI]	1.41	0.19	opaque
TiSE-5	1	0	7.8	1.25	1.0M LiTFSI/[PP14][TFSI]	0.96	0.21	opaque
SiSE-6	0	1	7.8	1.5	1.0M LiTFSI/[BMI][TFSI]	3.13	0.22	transparent
SiSE-7	0	1	7.8	1.5	0.6M LiTFSI/[Py14][TFSI]	1.20	0.24	transparent

Supplementary Table S2 The TiSE electrolytes with different molar ratio, and their phases and ILE content (wt%).

Phase	Molar ratios				ILE content (wt%)
	IL/Ti	Py13TFSI	Ti-nanogelator	HCOOH	
Solid	0	0	1	7.8	0
	0.25	0.25	1	7.8	61
	0.5	0.5	1	7.8	75
Quasi-solid	0.75	0.75	1	7.8	82
	1	1	1	7.8	86
	1.25	1.25	1	7.8	88
Liquid-like	1.5	1.5	1	7.8	90
	1.75	1.75	1	7.8	92
	2	2	1	7.8	93

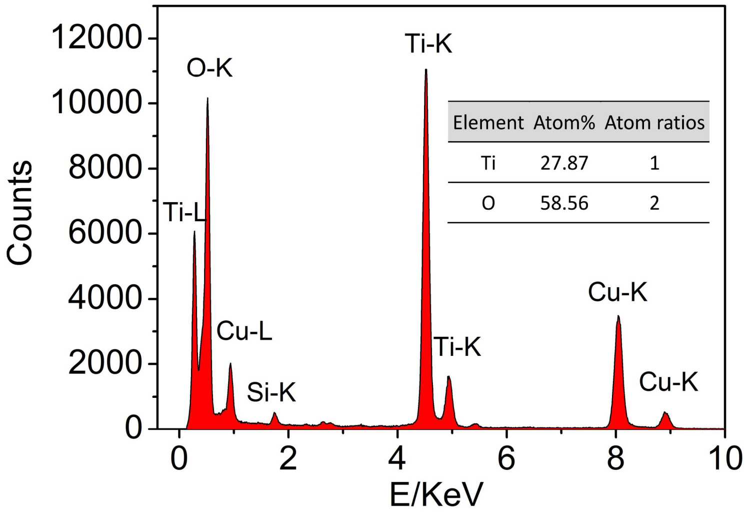

Element	Atom%	Atom ratios
Ti	27.87	1
O	58.56	2

Supplementary Figure S1 Energy dispersive spectra of the Ti-nanogelator matrix.

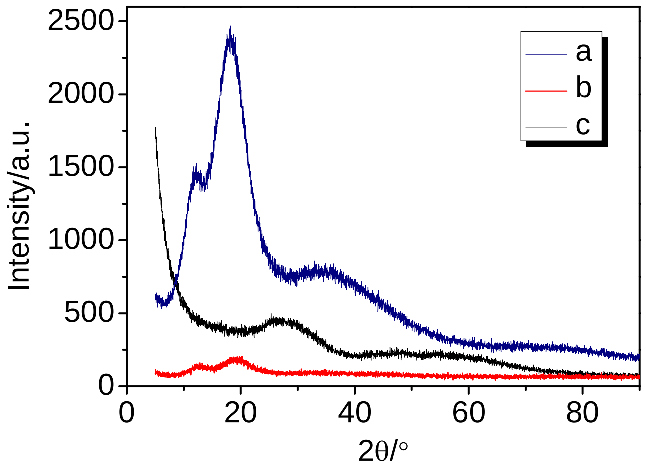

Supplementary Figure S2 XRD patterns of ionic liquid electrolyte a), TiSE-2 before b) and after c) removal of the ionic liquid.

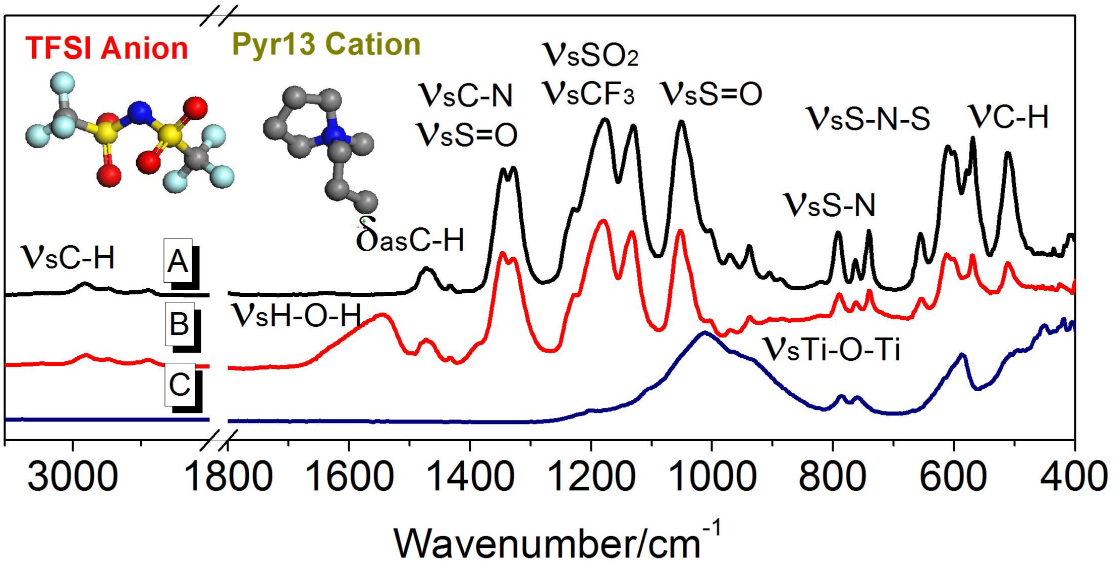

Supplementary Figure S3 IR spectra of ionic liquid electrolyte A), TiSE-2 monolith B), and Ti-nanogelator C). All characteristic peak of unconfined ILE can be found in the final TiSE-2 spectrum. Those results provide evidence of TiO_2 has been prepared and ILE network fully interconnected within the solid electrolyte.

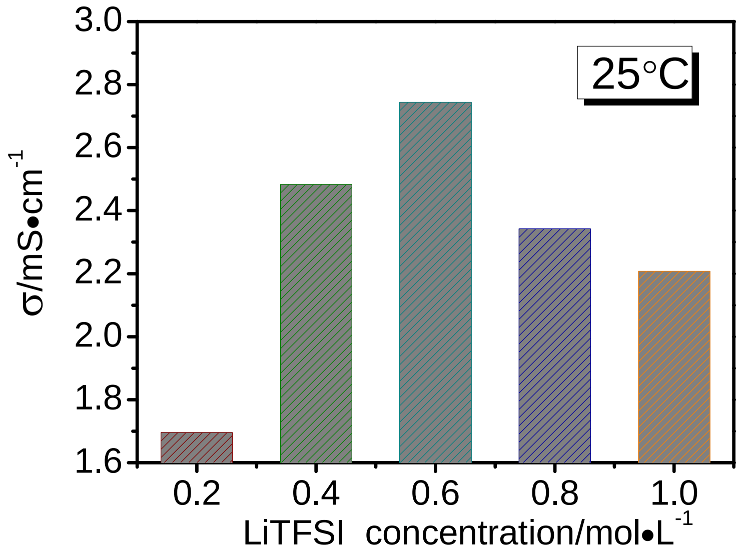

Supplementary Figure S4 The value of ionic conductivity of Py13-TFSI with 0.2, 0.4, 0.6, 0.8 and 1.0 M LiTFSI salt at room temperature.

Supplementary Table S3 Ionic conductivity of the nanogelator-based solid electrolytes.

Sample	Ionic Conductivity (Sm·cm^{-1})						
	-10°C	0°C	10°C	25°C	40°C	55°C	100°C
TiSE-1	0.42	0.77	1.39	2.74	4.6	8.2	23.1
TiSE-2	-	0.43	0.96	2.27	4.3	7.3	20.5
TiSE-3	0.12	0.32	0.72	1.82	3.5	6.2	16.5
TiSE-4	0.04	0.14	0.35	1.08	2.2	4.6	16.2
TiSE-5	0.02	0.06	0.19	0.72	1.7	3.5	13.4
SiSE-6	0.47	0.95	1.62	3.19	5.2	8.2	19.1
SiSE-7	0.01	0.03	0.14	0.47	1.1	2.2	10.9

Supplementary Table S4 VTF fitting parameters. A is assumed to be proportional to the number of carrier ions, B is the pseudo-activation energy, and T_0 is the reference temperature where the configurational entropy becomes zero.

Sample	VTF fitting parameters			R^2
	$A(Scm^{-1}K^{1/2})$	B(K)	T_0(K)	
TiSE-1	6.160±0.02	571±25	178±2	0.999
TiSE-2	2.604±0.03	657±20	186±2	0.999
TiSE-3	2.636±0.07	673±32	188±2	0.999
TiSE-4	3.049±0.06	775±36	188±3	0.999
TiSE-5	3.180±0.03	854±25	188±2	0.999
SiSE-6	2.099±0.07	622±44	171±2	0.999
SiSE-7	1.806±0.08	534±76	153±3	0.999

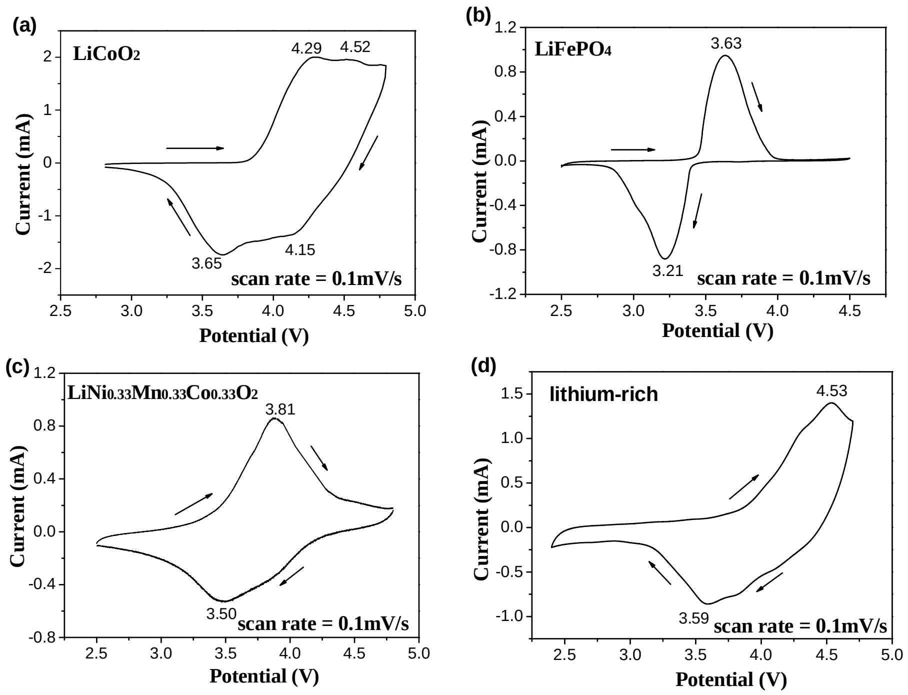

Supplementary Figure 5 Cyclic voltammograms of (a) Li/Py13–TiSE/$LiCoO_2$ cell, (b) Li/Py13-TiSE/ $LiFePO_4$, (c) Li/Py13-TiSE/ $LiNi_{0.33}Mn_{0.33}Co_{0.33}O_2$ cell and (d) Li/Py13-TiSE/lithium-rich cell.

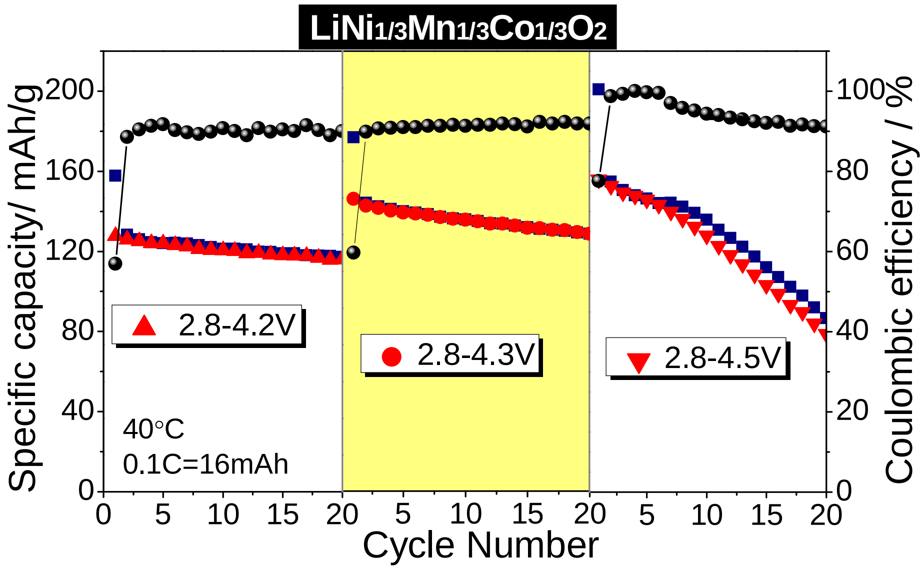

Supplementary Figure S6 Charge–discharge capacity during cycling of Li/ $LiNi_{0.33}Mn_{0.33}Co_{0.33}O_2$ half-cells in the range [2.8V–4.2V], [2.8V-4.3V] and [2.8V-4.5V] Vs. Li/Li^+, at C/10 rate and 40°C

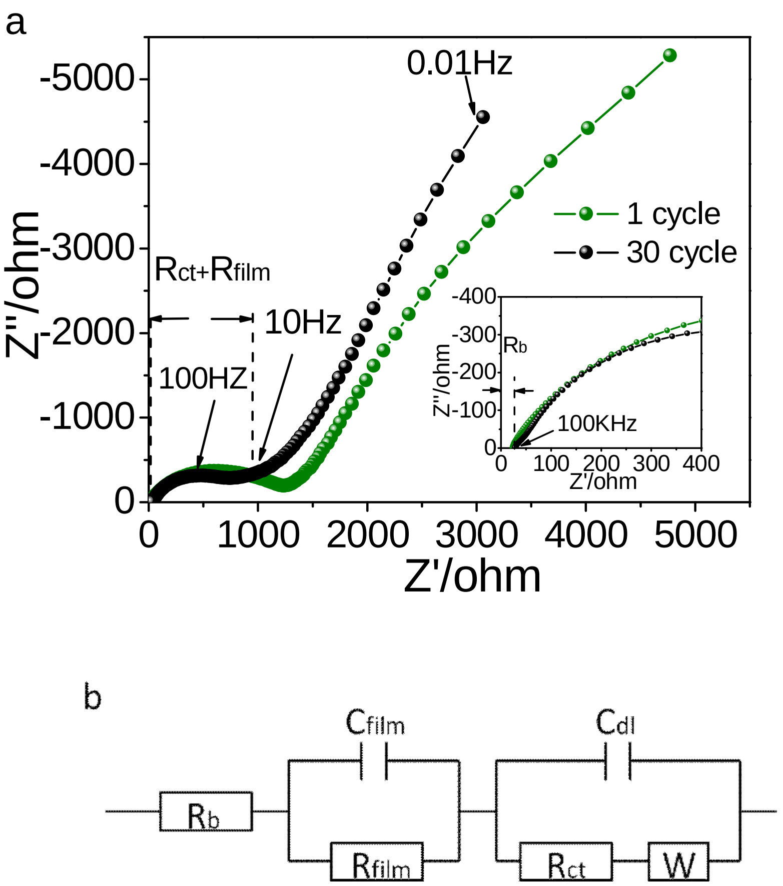

Supplementary Figure S7 a) Impedance plots of Li/Py13-TiSE/ lithium-rich cell before and after 30 cycles, the inset is a magnified view of the high frequency region of the impedance spectra. Frequency range: 100 KHz-10 mHz. b) Equivalent circuit model. The lower intersection of semicircles and the real axis in the impedance plots correspond to the bulk resistance (R_b) of a solid electrolyte layer. R_{film} and C_{film} are the resistance and capacitance of the passivating film. The diameter of the semicircle is attributed to the interfacial resistance (R_{ct}) between the electrode and the TiSE solid electrolyte. C_{dl} is the relative double-layer capacitance. The straight sloping line at low frequency represents the Warburg impedance (W).
